# On epidemiology and geographic information systems: a review and discussion of future directions.

**DOI:** 10.3201/eid0202.960202

**Published:** 1996

**Authors:** K C Clarke, S L McLafferty, B J Tempalski

**Affiliations:** Hunter College-CUNY, New York, New York 10021, USA. kclarke@everest.hunter.cuny.edu

## Abstract

Geographic information systems are powerful automated systems for the capture, storage, retrieval, analysis, and display of spatial data. While the systems have been in development for more than 20 years, recent software has made them substantially easier to use for those outside the field. The systems offer new and expanding opportunities for epidemiology because they allow an informed user to choose between options when geographic distributions are part of the problem. Even when used minimally, these systems allow a spatial perspective on disease. Used to their optimum level, as tools for analysis and decision making, they are indeed a new information management vehicle with a rich potential for public health and epidemiology.


### On Epidemiology and Geographic Information Systems: A Review and Discussion of Future Directions

**Keith C. Clarke, Ph.D., Sara L. McLafferty, Ph.D,. and Barbara J. Tempalski**
Hunter College-CUNY, New York, New York, USA

Geographic information systems are powerful automated systems for the capture, storage, retrieval, analysis, and display of spatial data. While the systems have been in development for more than 20 years, recent software has made them substantially easier to use for those outside the field. The systems offer new and expanding opportunities for epidemiology because they allow an informed user to choose between options when geographic distributions are part of the problem. Even when used minimally, these systems allow a spatial perspective on disease. Used to their optimum level, as tools for analysis and decision making, they are indeed a new information management vehicle with a rich potential for public health and epidemiology.

Geographic information systems (GIS) are "automated systems for the capture, storage, retrieval, analysis, and display of spatial data" (1). Common to all GIS is a realization that spatial data are unique because their records can be linked to a geographic map. The component parts of a GIS include not just a database, but also spatial or map information and some mechanism to link them together. GIS has also been described as the technology side of a new discipline, geographic information science (2), which in turn is defined as "research on the generic issues that surround the use of GIS technology, impede its successful implementation, or emerge from an understanding of its potential capabilities." Recently, GIS has emerged as an innovative and important component of many projects in public health and epidemiology, and this disciplinary crossover is the focus of this review.

Few would argue that GIS has little to offer the health sciences. On the other hand, like other new technologies, GIS involves concepts and analytic techniques that can appear confusing and can lead to misunderstanding or even overselling of the technology. In this article, we attempt to bridge the gaps between the principles of geographic information science, the technology of GIS, the discipline of geography, and the health sciences. Our intent is to introduce to the epidemiologist a set of methods that challenge the "visual" half of the scientist's brain.

Address for correspondence: Keith C. Clarke, Ph.D., Department of Geology and Geography, Hunter College-CUNY, 695 Park Avenue, New York, NY 10021, USA.;fax: 212-772-5268; e-mail: kclarke@everest.hunter.cuny.edu.

Computers were first applied to geography as analytical and display tools during the 1960s (3). GIS emerged as a multidisciplinary field during the 1970s. The discipline's heritage lies in cartography's mathematical roots: in urban planning's map overlay methods for selecting regions and locations based on multiple factors (4); in the impact of the quantitative revolution on the discipline of geography; and in database management developments in computer science.

Several factors combined in the 1970s to reinforce GIS development. First, computers became more accessible and less costly. Second, mainframe computers gave way to minicomputers and then workstations, which gave great power to the user and included the access to networks that has led to its own revolution in technology. Third, the types of user interface required to operate technical software changed from batch, command-line, and remote access to windowing systems and "point and click" graphic interaction. What had been expensive, slow, and difficult has rapidly become inexpensive, fast, and easy to use. A final but essential precondition to GIS development was the broad availability of public domain digital map data, in the form of maps of the landscape from the U.S. Geologic Survey and for census areas from the U.S. Census Bureau. The current GIS World Sourcebook (5) lists hundreds of system suppliers and sources of information and catalogs system capabilities. In short, GIS has now come of age, to the extent that the contributions of a growing number of parallel disciplines have both influenced and been influenced by GIS. Other disciplines now affecting GIS include forestry, transportation planning, emergency services delivery, natural hazards planning, marketing, archeology, surveying, and criminal justice. A wide array of capabilities and information awaits the health scientist ready to pursue an interest in GIS.

In this article, we consider the functional capabilities of GIS and how they can relate to epidemiology. We then review studies in epidemiology and health science where GIS has already made a contribution and introduce the technologic and analytic background. We review spatial analytic methods and concepts of use in epidemiology and conclude by examining what the near future holds for technologic changes and what these changes mean for the study of emerging infectious diseases and other health applications.

### GIS Functional Capabilities

GIS definitions usually focus on what tasks a GIS can do rather that what it is. GIS functional capabilities follow the standard GIS definitions; therefore, GIS can bring together the elements necessary for problem solving and analysis.

Data capture implies that 1) data can be input into the GIS from existing external digital sources; this is particularly the case when no data exist for a project, and the base data must be assembled from other studies, public domain datasets, and images. This usually means that GIS must be able to import the most common data formats both for image-type (raster) and line-type (vector) maps. 2) GIS can capture new map data directly; this means either that the user can scan the map and input it into the GIS or trace over a map's features using a digitizing tablet and enter them into the GIS map database. 3) The GIS can accomplish everything that a regular database system can, such as enter and edit data and update information in the existing database.

Data storage implies storage of both map and attribute data. Attribute data are usually stored in a relational database management system contained within the GIS and accessed by a spreadsheet or query-driven user interface. For storage, map data must be encoded into a set of numbers so that the geometry of the map is available for query, but also so that the map is stored digitally in one or more files. Image maps are usually stored as gridded arrays. Line maps are encoded by any one of several systems, but usually by using both the coordinate information and encoded topology, so that the relationships between points, lines, and areas, such as the adjacency of regions or the connectedness of lines, are known in advance. The more efficient and flexible these data formats or structures, the more operations can be performed on the map data without further processing.

Data records in GIS can be retrieved in one of two ways. The relational database manager allows searching, reordering, and selecting on the basis of a feature's attributes and their values. For example, the user may wish to select out and order alphabetically the names of all health clinics that had positive results in more than 10% of their tests. GIS also allows spatial retrieval. The user could select all clinics by region, by their latitude, or by their distance from the capital. The user could also select all clinics that are more than 10 km from a major road and within 100 m of a river or lake. In addition, combining searches is possible. There could be several data "layers," for example vegetation, rivers, transportation, and population of villages. A single retrieval could combine data from each of these layers in a single query. Layers can also be weighted, so that rivers, for example, are twice as important as roads in selecting villages with a population under 500 surrounded by forest.

Display functions include predominantly the making of maps. Tools must exist for constructing many types of maps, such as contours, symbols, shading or choropleth, and sized symbols. Formal map display often follows a series of more temporary map images, usually without a strict map composition, and the result of a test, an analysis, or a query. In addition, the GIS must be able to output finished format of maps to a medium, such as PostScript, on a plotter or printer, or onto photographic film.

Many tools exist to support field data collection. Tasks in which ancillary demographic information needs to be input and coregistered are simple. Habitat associated with a vector (e.g., a snail or a mosquito) may need remotely sensed data, such as vegetation cover or weather data. If these data are georegistered, integration is possible. One of the most useful functions is called address matching, in which street addresses with house numbers and street names are automatically placed into an administrative unit or placed as a dot on the map. Thus a digital phone list or mailing list of patients can be merged with the remainder of the data. In the United States, the Census Bureau's TIGER files can usually match 70% to 80% of unedited address records, and higher percentages if the address files are proofed and/or the more detailed and up-to-date commercial street files are used. In some field projects, the GIS's ability to make maps became the mainstay of the effort, allowing planning of truck and jeep routes, sequencing field clinics for optimal routes for visits, and even for local navigation. The ability to display maps often goes far beyond their final or use in the laboratory. Often a GIS image map is more accurate and up to date than anything available locally.

### Existing Applications of GIS in Epidemiology

Epidemiologists have traditionally used maps when analyzing associations between location, environment, and disease (6). GIS is particularly well suited for studying these associations because of its spatial analysis and display capabilities. Recently GIS has been used in the surveillance and monitoring of vector-borne diseases (7-9) water borne diseases (10), in environmental health (11-13), modeling exposure to electromagnetic fields (14), quantifying lead hazards in a neighborhood (15), predicting child pedestrian injuries (12), and the analysis of disease policy and planning (16).

In a recent study in Baltimore County, Maryland, GIS and epidemiologic methods were combined to identify and locate environmental risk factors associated with Lyme disease (7). Ecologic data such as watershed, land use, soil type, geology, and forest distribution were collected at the residences of Lyme disease patients and compared with data collected at a randomly selected set of addresses. A risk model was generated combining both GIS and logistic regression analysis to locate areas where Lyme disease is most likely to occur.

GIS allows analysis of data generated by global positioning systems (GPS). Combined with data from surveillance and management activities, GIS and GPS provide a powerful tool for the analysis and display of areas of high disease prevalence and the monitoring of ongoing control efforts. The marrying of GIS and GPS enhances the quality of spatial and nonspatial data for analysis and decision making by providing an integrated approach to disease control and surveillance at the local, regional, and/or national level.

GIS is being used to identify locations of high prevalence and monitor intervention and control programs in areas of Guatemala for onchocerciasis (9) and in Africa for trypanosomiasis (17). Spatial and ecologic data are combined with epidemiologic data to enable analysis of variables that play important roles in disease transmission. This integration of data is essential for health policy planning, decision making, and ongoing surveillance efforts. For example, as part of the guinea worm eradication effort, the United Nation's Children's Emergency Fund placed pumps in villages most infected with the disease to ensure access to a safe water supply (18). GIS enabled researchers to locate high prevalence areas and populations at risk, identify areas in need of resources, and make decisions on resource allocation (16). Epidemiologic data showed a marked reduction in prevalence in villages where pumps were introduced.

GIS was used in designing a national surveillance system for the monitoring and control of malaria in Israel (19). The system included data on the locations of breeding sites of *Anopheles* mosquitoes, imported malaria cases, and population centers. The GIS-based surveillance system provided means for administrative collaboration and a network to mobilize localities in the case of outbreaks.

In 1985, the National Aeronautics and Space Administration (NASA) established the Global Monitoring and Disease Prediction Program at Ames Research Center in response to the World Health Organization's call for the development of innovative solutions to malaria surveillance and control (20). A major aspect of the program was to identify environmental factors that affect the patterns of disease risk and transmission. The overall goal of the program was to develop predictive models of vector population dynamics and disease transmission risk using remotely sensed data and GIS technologies.

Remotely sensed data have been used in many vector disease studies (8,17,21-24). Remote sensing and GIS were used to identify villages at high risk for malaria transmission in the southern area of Chiapas, Mexico (8). An earth environmental analysis system for responding to fascioliasis on Red River Basin farms in Louisiana was developed by integrating LANDSAT MSS imagery with GIS (22). In Kwara State, Nigeria, a temporal analysis of Landsat Thematic Mapper (TM) satellite data was used to test the significance of the guinea worm eradication program based on changes in agricultural production (21).

### Spatial Analysis and GIS

GIS applications show the power and potential of such systems for addressing important health issues at the international, national, and local levels. Much of that power stems from the systems' spatial analysis capabilities, which allow users to examine and display health data in new and highly effective ways. Spatial analysis refers to the “ability to manipulate spatial data into different forms and extract additional meaning as a result” (25). It encompasses the many methods and procedures, developed in geography, statistics, and other disciplines, for analyzing and relating spatial information. Spatial relationships, those based on proximity and relative location, form the core of spatial analysis.

Gatrell and Bailey (26) describe three general types of spatial analysis tasks: visualization, exploratory data analysis, and model building. These range in complexity from simple map overlay operations to statistical models such as spatial interaction and diffusion models. The value of maps for public health analysis has long been recognized; John Snow’s now classic maps of cholera cases in relation to the Broad Street pump are a good example. However, with its extensive data management and display capabilities, GIS offers much more than simple mapping. Map overlay operations allow the analyst to compute new values for locations based on multiple attributes or data “layers” and to identify and display locations that meet specific criteria (27). For example, in targeting locations for mosquito vector control, one might want to identify areas that have low elevation, specific types of vegetation favored by mosquitoes, and are within 100 m of ponds or other water bodies. Each of these attributes comprises a distinct data layer. With GIS, one can create 100-m buffers around water bodies and then select areas meeting all three criteria. Display of these areas on a GIS-generated map has obvious benefits for planning vector control strategies.

As indicated previously, this general class of procedures for weighing and overlaying maps, also known as “suitability analysis,” has been used in diverse health applications. Typically the criteria and weights attached to them are specified by the analyst based on expert knowledge or prior research. Using the computational and visual display capabilities of GIS, one can then explore the sensitivity of results to the weights and cutoff values used. Another approach is to employ regression analysis to generate the linear combination of factors that best explain spatial variation in disease prevalence. The weights from the regression model are used to create a composite index of risk which can then be mapped (7).

Visualization is also an important tool for showing the change in disease patterns over time. Animation, embedded within a GIS, is highly effective in depicting the spread or retreat of disease over space and time. A series of animated maps were created to show the advance of the AIDS epidemic in the United States as it moved from and within major cities (28). One could imagine a similar animated map sequence showing the retreat and eventual eradication of a disease like smallpox. Clearly much more research is needed in this area, especially research that links animation to theoretical models of disease diffusion, within a GIS environment.

Visualization can be used in novel ways to explore the results of traditional statistical analysis. Displaying the locations of outlier and influential values on maps and showing variation in values over space can add a great deal to epidemiologic research. Although such tools are being developed and explored, they would benefit greatly from a closer and more seamless link between statistical packages and GIS (25).

The second general class of GIS methods addresses exploratory spatial analysis. These methods allow the analyst to sift meaningfully through spatial data, identify “unusual” spatial patterns, and formulate hypotheses to guide future research (26). The quantity and diversity of spatial data in GIS can be overwhelming: exploratory methods help the analyst make sense of data and address “what if” questions. Advances in computing and graphics technology have made this one of the most active areas in GIS/spatial analysis research.

Among the most important exploratory methods for epidemiology and public health are methods for identifying space-time clusters or “hot spots” of disease. Openshaw’s geographic analysis machine (GAM) was an early method that worked completely within a hybrid GIS. The GAM’s many applications included an attempt to determine if spatial clusters of childhood leukemia were located near nuclear facilities in Britain (29). The GAM works with point data on disease cases and searches at regular intervals for statistically significant clusters of disease prevalence. Maps display the locations of significant clusters, showing the proximity of clusters to hypothesized environmental threats such as nuclear facilities. Although Openshaw’s work was widely criticized on statistical grounds, it opened the door for an active body of research on exploratory spatial analysis of disease. Some of the new methods that have been developed as outgrowths of Openshaw’s approach have been published (30).

Exploratory methods are also valuable in searching for zones or districts of high disease prevalence. Because areas may differ greatly in population size, prevalence rates have different levels of variability and thus reliability (31). Researchers have long used probability mapping to show the statistical significance of prevalence rates (32); however, probability mapping does not give a sense of the actual rates or the populations on which they are based. An alternative method is to smooth rates towards a regional or local mean value using empirical Bayes methods (33). Although GIS and empirical Bayes methods have developed separately, there is much scope for interaction. For example, GIS can be used to generate geographically based regional or local means to which actual rates are smoothed. These might be based on averaging rates for contiguous areas (33,34); or they might rely on more complex, multivariate, spatial clustering procedures that incorporate proximity as well as population attributes.

Many methods for exploratory analysis of disease patterns are not appropriate for infectious diseases because the methods are essentially static and assume independence. For infectious diseases, cases clearly are not independent and the diseases move through time and space. In these situations, one can use spatial autocorrelation methods and space-time correlograms to explore the spatial and temporal patterns of infectious disease spread (35).

These methods provide a general sense of the speed and geographic pattern of disease transmission. Although the methods have not typically been incorporated in GIS, there is great potential for doing so, especially with recent advances in computer animation.

Modeling, the final class of spatial analysis methods, includes procedures for testing hypotheses about the causes of disease and the nature and processes of disease transmission. In general, modeling involves the integration of GIS with standard statistical and epidemiologic methods. GIS can assist in generating data for input to epidemiologic models, displaying the results of statistical analysis, and modeling processes that occur over space. The first two points are evident in recent, regression-based analyses of disease risk, such as the study of Lyme disease (7). There GIS was used not only to integrate diverse datasets and calculate new variables, such as slope and distance from forest, but also to map geographic variation in disease risk, as predicted from a logistic regression model.

Other GIS models are more explicitly spatial, expressing relationships or flows between people and places. Spatial interaction and spatial diffusion models are of particular relevance to the study of emerging diseases. Spatial interaction models analyze and predict the movements of people, information, and goods from place to place (36). The flows of people between rural areas, villages, cities, and countries are all forms of spatial interaction that are central to disease transmission. By accurately modeling these flows, it is possible to identify areas most at risk for disease transmission and thus target intervention efforts. Spatial interaction models reflect two general principles: that interaction decreases with distance and increases with population size or "attractiveness." Given actual flow data, one can estimate values that show the effects of distance and population size (or other "attractiveness" factors) on interaction. The models can then be used to predict spatial interaction patterns elsewhere. Although spatial interaction models and GIS developed separately, some GIS now have spatial interaction modeling capabilities (37).

Spatial diffusion models analyze and predict the spread of phenomena over space and time and have been widely used in understanding spatial diffusion of disease (38). Such models are quite similar to spatial interaction models except that they have an explicit temporal dimension. By incorporating time and space, along with basic epidemiologic concepts, the models can predict how diseases spread, spatially and temporally, from infected to susceptible people in an area (39) and aid in understanding the emergence of infectious disease (40).

### Data

Important technical and logistic innovations in data and data access for GIS are under way and will come to fruition before the end of the century. First, and by far the most important, have been increased access to the Defense Department's global positioning systems (GPS), the availability of inexpensive hand-held devices for using the system, and the addition of direct-to-GIS data links to these systems. For a relatively modest investment, field users can add geographic coordinates to their data collection from anywhere in the world, at any time, and in any weather. These systems are so flexible that their antennas can be placed on top of a car, and the logger can be connected to a portable computer on the dashboard, so that as the user drives along, the path of the vehicle is permanently recorded in the GIS's own data format and displayed on screen with a 1-s update. As these systems have become more common, they have also gained in precision and accuracy. It is not uncommon for fixes to be corrected using a process known as differential GPS, either after the fact by computer software or in real time, so that each point is recorded to the nearest meter on the ground. GPS and GIS together have permanently altered the relationship between field data collection and data analysis. Data collected in real time can be analyzed the same day and acted upon immediately.

Similarly, various devices used for capturing overhead images and photographs have undergone a similar revolution. First, technology has improved, allowing images in the infrared, thermal, radar and other wavelengths to be collected at higher and higher spatial resolutions. Second, massive changes in policy have resulted from the end of the Cold War. Formerly secret satellite data, such as the CORONA and Russian spy imagery, are now broadly available, even searchable on the Internet. In the United States, the National Air Photo program intends to remap the country every 5 years at a scale of 1:12,000 with 1-m resolution and publish the images as CD-ROMs. In addition, NASA's largest ever Mission to Planet Earth and its Earth Observation System will begin to return unimaginable amounts of information about the whole earth's geography and atmosphere well before the end of the century. The data will be available to any Internet user and distributed by a set of active archive centers.

Third, technical issues related to data transfer have been partially eliminated. This has come about by the convergence toward sets of industry standard formats such as GIF and TIF for images and new national and international digital map data standards. In addition, efforts are now under way to standardize reference information about datasets, termed metadata, so that the equivalent of a Library of Congress cataloging will be possible.

Finally, many datasets have become available that can form at least the skeleton of a new GIS project almost anywhere in the world. By combining public domain datasets, such as the Digital Chart of the World and satellite imagery, with GPS and field data, the claim that data collection and changes in format constitute 80% of the effort in a GIS project is rapidly being eroded and replaced by a mere morning spent surfing the Internet. Nevertheless, many of the world's nations are still poorly mapped at the more detailed spatial scales required for local analysis.

### Hardware

GIS hardware has continued to improve. On the high end, workstations have both increased in power and dropped in price, making this platform the choice for large, laboratory-based GIS projects. As the GIS software packages have been modified for the workstation operating systems, most commonly UNIX with X-Windows, operations that were impossible because of computational complexity have now become commonplace. This trend will continue to the extent that few technical constraints like memory and central processing unit (CPU) power will exist for GIS. Some tasks, such as skilled visual image identification and interpretation, have been partly or wholly automated. On the low end, microcomputers have become immensely powerful and fast, easily capable of performing basic GIS operations even on portable computers. The theme of GIS mobility, added to satellite and cellular telephone communications, has permanently transformed the ability to operate with GIS in the field, and will lead to a new "data rich" era for epidemiologic study.

In addition, the next generation of systems will depend on network computing. Networks have allowed de facto parallel computing within a local area network. By supporting personal multitasking, they have allowed data to be held in a distributed way and retrieved for use on demand, and the network has built an immensely powerful support structure for information sharing. The World-Wide Web, for example, can deliver to a workstation-user free GIS software, data, and information on how to install and use the system, support for technical problems, and even an outlet to publish scientific results.

### Software

GIS software has improved remarkably in the latest generation and will undergo still more changes. The basic tools of the computer programmer have undergone a transition from first generation to object-oriented database and programming languages, offering some benefits in program module reusability, improved data handling, and ease of use as more and more packages are rewritten to take advantage of these tools. The WIMP (windows, icons, menus, and pointers) interfaces so common today owe their origins to this technology. Today, the GIS research community suggests that as the "desktop metaphor" becomes more commonly accepted, increasingly sophisticated metaphors will take over for organizing computing, including perhaps using maps themselves to manage the computer rather than vice versa.

Some changes are far more practical but still of great value. Most software systems now support context-sensitive help, electronic manuals, and automatic installation and update procedures. Each of these could benefit from intelligent software that uses an expert system base and continues to tailor the system around the GIS operator's revealed use. Such software, used over a network, has been termed an intelligent agent. Most GIS of the future will use these methods to seek out new data over the network that relate to your problem, alert you to mistakes in your data management and analysis, and perhaps automatically compose maps and reports at the completion of a project.

Multimedia and hypermedia are also rapidly becoming a component of GIS software. Multimedia allow simultaneous use of text, sound, animation, and graphics. GIS software has also developed the ability to interact in many spoken languages, under different operating systems, and on many different computers. The independence of the software and the tasks from particular computer platforms, or even vendors, are a highly desirable element in a distributed system.

### GIS and Public Health

While it holds distinct promise as a tool in the fight against emerging infectious diseases and other public health problems; it is not simply the next widget to come into play. GIS can be seen as a new approach to science, one with a history and heritage, a finite and well researched suite of methods and techniques, and a research agenda of its own. It does not fit neatly into the health scientist's toolbox. It requires rethinking and reorganizing the way that data are collected, used, and displayed. It requires expense, training, and a climb up a learning curve. It needs maintenance and support and can be both overwhelming and threatening to the uninitiated.

On the other hand, the base of research and scholarship using GIS in the health sciences cannot be ignored. A first step would be to integrate instruction on GIS into college curricula in public health. An admirable body of experience in GIS education already exists, even a thoroughly tested national curriculum that can be easily adapted to a new set of demands (41). A second step would be to seek out more formal links between the research communities working with GIS. There are astonishing similarities for example in the field requirements for using GIS between forestry, ecology, archeology and epidemiology that could provide substantial benefits by the sharing of experiences and the pooling of resources.

Above all, GIS should be seen as improving the set of tools to promote public health. Good epidemiologic science and good geographic information science go hand in hand. The future of GIS has already retained a role for the geographically literate public health expert. Epidemiologists should seize the opportunity to set their own agenda and influence the technology and science toward the goal of public health.

*Dr. Clarke is professor and chair, Department of Geology and Geography, Hunter College, and on the faculty of the Earth and Environmental Sciences Program at the Graduate School and University Center of the City University of New York. Dr. Clarke's most recent research has been on environmental simulation modeling, the impact of the Persian Gulf War on the technology of cartography, and mapping to support disease control programs in Africa.*

### References

1. Clarke KC. Analytical and computer cartography. 2nd ed. Englewood Cliffs, NJ: Prentice-Hall. 1995.
2. Goodchild MF. Geographical information science. International Journal of Geographical Information Systems 1992; 6(1).
3. Tobler WR. Automation and cartography. Geographical Review 1959;49:526-34.
4. Steinitz C, Parker P, Jordan L. Hand-drawn overlays: their history and prospective use. Landscape Architecture 1976;66:444-55.
5. Geographic Information Systems. GIS world sourcebook. Fort Collins, CO: GIS World, Inc., 1995.
6. Gesler W. The uses of spatial analysis in medical geography: a review. Soc Sci Med 1986;23:963-73.
7. Glass GE, Schwartz BS, Morgan JM III, Johnson DT, Noy PM, Israel E. Environmental risk factors for Lyme disease identified with geographic information systems. Am J Public Health 1995;85:944-8.

8. Beck LR, Rodrigues MH, Dister SW, Rodrigues AD, Rejmankova E, Ulloa A, et al. Remote sensing as a landscape epidemiologic tool to identify villages at high risk for malaria transmission. Am J Trop Med Hyg 1994;51:271-80.
9. Richards FO, Jr. Use of geographic information systems in control programs for onchocerciasis in Guatemala. Bull Pan Am Health Organ 1993;27:52-5.
10. Clarke KC, Osleeb JR, Sherry JM, Meert JP, Larsson RW. The use of remote sensing and geographic information systems in UNICEF's dracunculiasis (Guinea worm) eradication effort. Prev Vet Med 1991;11:229-35.
11. Cuthe WG, Tucker RK, Murphy EA, England R, Stevenson E, Luckardt JC. Reassessment of lead exposure in New Jersey using GIS technology. Environ Res 1992;59:318-25.
12. Braddock M, Lapidus G, Cromley E, Cromley R, Burke G, Branco L. Using a geographic information system to understand child pedestrian injury. Am J Public Health 1994;84:1158-61.
13. Barnes S, Peck A. Mapping the future of health care: GIS applications in Health care analysis. Geographic Information systems 1994;4:31-3.
14. Wartenberg D, Greenberg M, Lathrop R. Identification and characterization of populations living near high-voltage transmission lines: a pilot study. Environ Health Perspect 1993;101:626-32.
15. Wartenberg D. Screening for lead exposure using a geographic information system. Environ Res 1992 Dec;59:310-7.
16. Tempalski BJ. The case of Guinea worm: GIS as a tool for the analysis of disease control policy. Geographic Information Systems 1994;4:32-8.
17. Roger DJ, Williams BG. Monitoring trypanosomiasis in space and time. Parasitology 1993; 106(Suppl):277-92.
18. World Health Organization. Dracunculiasis: global surveillance summary, 1989. WHO Bull 1990;68:797-8.
19. Kitron U, Pener H, Costin C, Orshan L, Greenberg Z, Shalom U. Geographic information system in malaria surveillance: mosquito breeding and imported cases in Israel, 1992. Am J Trop Med Hyg 1994;50:550-6.
20. Wood BL, Beck LR, Dister SW, Spanner MA. Global monitoring and disease prediction program. Submitted January 1994. Sistema Terra.
21. Ahearn SC, De Rooy C. Monitoring the effects of dracunculiasis remediation of agricultural productivity using satellite data. Accepted for publication 1996. International Journal of Remote Sensing.
22. Malon JB, Fehler DP, Loyacano AF, Zukowski SH. Use of LANDSAT MSS imagery and soil type in a geographic information system to assess site-specific risk of fascioliasis on Red River Basin farms in Louisiana. Ann NY Acad Sci 1992;652:389-97.
23. Washino RK, Wood BJ. Application of remote sensing to arthropod vector surveillance and control. Am J trop Med Hyg 1994;50(6 Suppl):134-44.
24. Zukowski SH, Wilkerson GW, Malone JB, Jr. Fasciolosis in cattle in Louisiana. II. Development of a system to use soil maps in a geographic information system to estimate disease risk on Louisiana coastal marsh rangeland. Vet Parasitol 1993;47:51-65.
25. Bailey, T. A review of statistical spatial analysis in geographical information systems. In: Fotheringham S, Rogerson P. Spatial analysis and GIS. London: Taylor and Francis. 1994.
26. Gatrell A, Bailey T. Can GIS be made to sing and dance to an epidemiological tune? Presented at the International Symposium on Computer Mapping and Environmental Health, Tampa, FL, February 1995.
27. Tomlin WR. Geographic information systems and cartographic modelling. Englewood Cliffs, NJ: Prentice-Hall, 1990.
28. Gould P. The slow plague: a geography of the AIDS epidemic. Cambridge, UK: Blackwell, 1993.
29. Openshaw S, Charlton M, Wymer C, Craft A. A mark 1 geographical analysis machine for the automated analysis of point data sets. International J Geographical Information Systems 1987;1:335-58.
30. Marshall R. A review of methods for the statistical analysis of spatial patterns of disease. J R Stat Soc 1991;154:421-41.
31. Cressie N. Smoothing regional maps using empirical Bayes predictors. Geographical Analysis 1992;24:75-95.
32. Choynowski M. Maps based upon probabilities. J Am Stat Assoc 1959;54:385-8.
33. Clayton D, Kaldor J. Empirical Bayes estimates of age-standardized relative risks for use in disease mapping. Biometrics 1987;43:671-87.
34. Ord K, Getis A. Local spatial autocorrelation statistics: distributional issues and an application. Geographical Analysis 1995;24:286-306.
35. Cliff A, Haggett P. Atlas of disease distributions: analytic approaches to epidemiological data. Oxford; UK: Blackwell Reference, 1988.
36. Haynes K, Fotheringham AS. Gravity and spatial interaction models. Beverly Hills, CA: Sage, 1984.
37. Ding Y, Fotheringham AS. The integration of spatial analysis and GIS. Computers in Environmental and Urban Systems 1992:3-19.
38. Cliff A, Haggett P, Smallman-Raynor, M. Measles: an historical geography of major human viral disease from global expansion to local retreat. Oxford, UK: Blackwell Reference, 1993.
39. Thomas R. Geomedical systems: intervention and control. New York: Routledge, 1990.
40. Haggett P. Geographical aspects of the emergence of infectious diseases. Geografiska Annaler, B 1994;76:91-104.
41. National Center for Geographic Information and Analysis. NCGIA Core Curriculum in GIS, 1990. URL: http://www.ncgia. uscb.edu/pubs/core.html.
